# Specific eukaryotic plankton are good predictors of net community production in the Western Antarctic Peninsula

**DOI:** 10.1038/s41598-017-14109-1

**Published:** 2017-11-01

**Authors:** Yajuan Lin, Nicolas Cassar, Adrian Marchetti, Carly Moreno, Hugh Ducklow, Zuchuan Li

**Affiliations:** 1 Division of Earth and Ocean Sciences, Nicholas School of the Environment, Duke University, Durham, NC 27708 USA; 2 Department of Marine Sciences, University of North Carolina at Chapel Hill, Chapel Hill, NC 27514 USA; 3 Lamont-Doherty Earth Observatory, Columbia University, Palisades, NY 10964 USA; 4 Université de Brest - UMR 6539 CNRS/UBO/IRD/Ifremer, Laboratoire des sciences de l’environnement marin – IUEM, Rue Dumont D’Urville, Plouzané, 29280 France

## Abstract

Despite our current realization of the tremendous diversity that exists in plankton communities, we have little understanding of how this biodiversity influences the biological carbon pump other than broad paradigms such as diatoms contributing disproportionally to carbon export. Here we combine high-resolution underway O_2_/Ar, which provides an estimate of net community production, with high-throughput 18 S ribosomal DNA sequencing to elucidate the relationship between eukaryotic plankton community structure and carbon export potential at the Western Antarctica Peninsula (WAP), a region which has experienced rapid warming and ecosystem changes. Our results show that in a diverse plankton system comprised of ~464 operational taxonomic units (OTUs) with at least 97% 18 S identity, as few as two or three key OTUs, i.e. large diatoms, Phaeocystis, and mixotrophic/phagotrophic dinoflagellates, can explain a large majority of the spatial variability in the carbon export potential (76–92%). Moreover, we find based on a community co-occurrence network analysis that ecosystems with lower export potential have more tightly coupled communities. Our results indicate that defining plankton communities at a deeper taxonomic resolution than by functional groups and accounting for the differences in size and coupling between groups can substantially improve organic carbon flux predictions.

## Introduction

The biological carbon pump, fueled by nutrient delivery to the sunlit layers of the ocean, is believed to be a function of multiple processes, including net community production (NCP), where NCP is equal to gross primary production minus community respiration. Plankton communities regulate NCP through their influence on the fate of organic matter production, which can be either recycled or exported to depth depending on phytoplankton size and density^1–3^, as well as microbial and grazing activities^4^. This is generally predicted and modeled to be a function of Plankton Functional Types (PFTs). PFTs refer to collections of organisms based on explicit biogeochemical roles or requirements. Conventional PFTs include picoautotrophs, diazotrophs (N_2_-fixers), calcifiers (e.g., coccolithophorids), and silicifiers (e.g., diatoms)^5–7^. These PFTs are expected to contribute differently to carbon export production and carbon export efficiency. For example, most diatoms are believed to be efficient carbon exporters to depth because of their large size and heavy silica shells, which make them sink faster^8^.

In this study, we combine high-frequency NCP estimates^9,10^ with in-depth molecular taxonomic measurements^11–13^ to explore the relationship between the carbon export potential and plankton community assemblages at the Western Antarctic Peninsula (WAP). High-throughput sequencing has recently been shown to be a powerful tool to explore the influence of plankton community structure on carbon export in the oligotrophic oceans^14^. We apply similar molecular tools at the WAP, where large spatial variability in iron and light availability drives steep gradients in plankton diversity and NCP^15,16^. Furthermore, the WAP may represent the “canary in a coal mine” of the Southern Ocean, a region that has a disproportionate influence on global climate^17^. The WAP region has experienced drastic changes^18,19^ over the last 50 years warming at an unprecedented rate^20^. Community production at the bottom of the food chain, and local and remote upper trophic levels (e.g., krill, fish, penguins and other seabirds, seals, and whales) are likely to be affected by this rapid warming and associated sea ice loss^21^.

**Figure 1:**
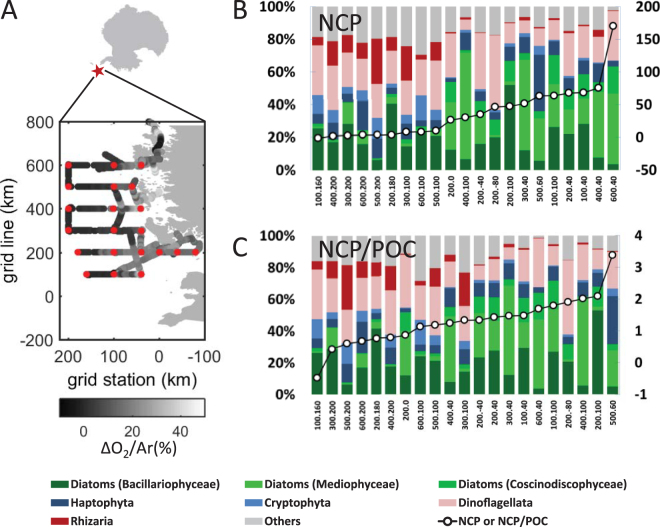
Eukaryotic plankton community composition of major taxonomic groups versus NCP. (**A**) Location of the Western Antarctica Peninsula and sampling sites (red dots) projected on Palmer LTER grid (x axis – grid station, y axis – grid line, in km). Samples were taken across a large gradient in biological O2. Stations (labeled as “grid line. grid station”) are ranked in ascending values of NCP (black circles) (**B**) in mmol C m^−2^ day^−1^, and (**C**) NCP/POC in m day^−1^. The relative abundance of different taxa (Phylum to Division) in % of 18 S reads are represented in different colors. Centric diatoms (Mediophyceae and Coscinodiscophyceae) are in light greens, whereas pennate diatoms (Bacillariophyceae) are in dark green. Less dominant taxa (<5% in total abundance) are combined into ‘others’ in grey. The maps in this figure are generated by MATLAB R2014a (http://www.mathworks.com/).

Using DNA sequencing, we first characterized community structure at a taxonomic level comparable to PFTs and High Performance Liquid Chromatography (HPLC) pigment-based definitions (Fig. 1, Supplementary Table S1). At this level of phylum to division, the sequences were grouped into diatoms, Haptophyta, Cryptophyta, Dinoflagellata, among others. Based on relative contributions, we found that diatoms were the dominant components of the plankton population during our survey, accounting for an average of 41% of the total reads. In order to account for the influence of biomass on NCP, we normalized NCP to Particulate Organic Carbon (POC) concentration in the mixed layer to identify “High Biomass, Low NCP” and “Low Biomass, High NCP” regime^10,22^. Cassar *et al*. (2015)^10^ hypothesized based on theoretical considerations that the POC-normalized NCP may, under some conditions, provide insight into the apparent sinking rate and turn over time of the organic carbon inventory in the mixed layer. We found that the relative abundance of diatoms was positively correlated with NCP (r^2^ = 0.35, *p* = 0.005) but that there was no significant correlation with NCP/POC. Diatoms were further divided into centrics and pennates. Near the coast, on the continental shelf, and in the open ocean, the relative abundance (%) of centrics averaged 36.8 ± 16.2, 22.9 ± 26.5, and 5.6 ± 4.7; whereas pennates averaged 14.2 ± 8.2, 23.9 ± 15.5, and 22.2 ± 11.9, respectively. While there were no significant offshore-onshore trends in the relative abundance of pennate diatoms, the relative abundance of centric diatoms was significantly higher near the coast compared to the open ocean (non-parametric Mann-Whitney U test, P < 0.01). A positive correlation was observed between the relative abundance of centric diatoms and NCP (r^2^ = 0.47, *p* = 0.0006), as well as centric diatoms and NCP/POC (r^2^ = 0.20, *p* = 0.04). Surprisingly, the relative abundance of pennate diatoms was not significantly correlated with NCP or NCP/POC, which may be explained by the difference in size between centrics and pennates in the region (see discussion below). However, should this relation hold in other regions of the Southern Ocean, a correlation between HPLC pigment estimates of total diatom abundance and NCP may not be observed. This could explain why earlier studies found no clear correlation between HPLC diatom relative abundance and NCP in the WAP region^15^ and in the Subantarctic Zone south of Australia^10^.

**Figure 2:**
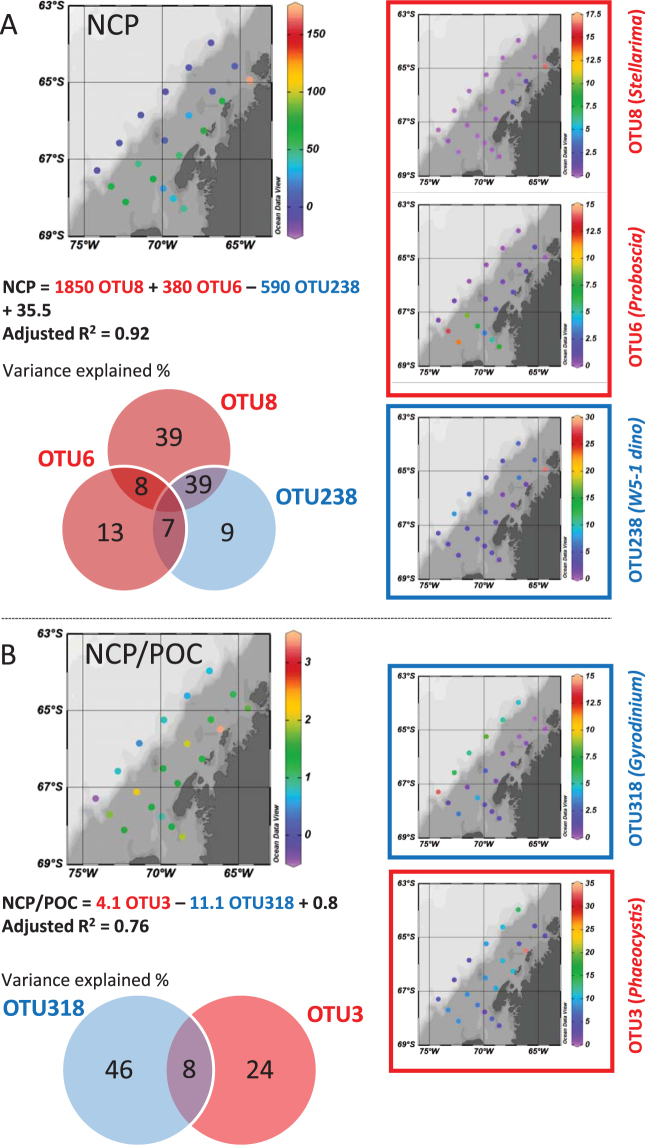
Community composition at the OTU (97%) level explaining carbon export. In a multiple linear regression model, 3 and 2 key OTUs selected by SSVS explained most of the variance in (**A**) NCP in mmol C m^−2^ day^−1^ and (**B**) NCP/POC in m day^−1^ geographical variability, respectively. Each OTU (relative 18 S abundance in %) has distinct biogeography, and its variance partitioning is shown in a Venn diagram. OTUs in red (blue) are positively (negatively) correlated with NCP or NCP/POC. Maps are created with Ocean Data View version 4.6.3.1 (Schlitzer, R., Ocean Data View, http://odv.awi.de, 2015).

Little is known about the carbon export potential of particular eukaryotic plankton species. The vast array of plankton attributes (e.g., cell biovolume, elemental ratios, and degree of silicification for diatoms) and the associated food web dynamics (grazing, aggregation, and fecal pellet export) are likely to be first-order controls on the carbon export potential. In order to gain insight into how NCP relates to the presence of specific eukaryotic plankton, we further characterized plankton assemblages at the OTU level (i.e., species to genus) based on a 97% DNA similarity threshold (Fig. 2). In total, 464 OTUs were defined in the surveyed region. The top 20 OTUs, a small portion (4.3%) of the total diversity, accounted for 78% of the overall sequence read abundance. This is consistent with the hyperdominance of few taxa in oceanic plankton ecosystems as recently reported by the Tara Oceans plankton survey^11^. Based on the relative abundance of these 20 OTUs, a stochastic search variable selection (SSVS) analysis identified which OTUs were the best predictors of NCP and NCP/POC (Supplementary Table S2). Stepwise regression analyses confirmed the top variables identified by SSVS (Supplementary Table S3). In the resulting multiple linear regression model based on the SSVS selected variables, three OTUs accounted for 92% of the NCP variance (Fig. 2A, Supplementary Table S3), and only two OTUs explained 76% of the NCP/POC variance (Fig. 2B, Supplementary Table S3). Both are significantly higher than the variance explained by the relative abundance at the higher taxonomic level (Supplementary Table S1), e.g. bulk diatoms explained 35% and 16% of the NCP and NCP/POC variances, respectively. Interestingly, the relative abundance of the most dominant OTU (OTU_4) (Supplementary Fig. S2), a pennate diatom belonging to the genus *Fragilariopsis*, was not a good predictor of the geographic variability of NCP (r^2^ = 0.12, *p* > 0.1) or NCP/POC (r^2^ = 0.05, *p* > 0.1). Many *Fragilariopsis* species are ice algae and the high abundance in some areas may be due to sea ice melt seeding rather than new cell growth. Our results suggest that PFTs may not be good predictors of NCP in some regions and that the intra-PFT variability may be greater than inter-PFT in terms of NCP.

The relative abundances of two diatoms with distinct biogeography, i.e. *Stellarima* (OTU_8) in a north bloom near Palmer Station and *Proboscia* (OTU_6) dominating the south ice edge bloom (Fig. 2; Supplementary Fig. S1), were both positively correlated with NCP but not NCP/POC. This suggests that these OTUs are associated with high production but relatively less efficient export systems. In contrast, the haptophyte *Phaeocystis* (OTU_3) was not correlated with NCP, but positively correlated with NCP/POC. *Phaeocystis* accounted for only 9.0% of the total 18 S reads on average, but co-dominated (34.4%) with diatoms (36.1%) at station (600.040) that had the highest NCP/POC (Fig. 2). *Phaeocystis* has been reported to be a major contributor to high particle flux out of the euphotic zone in the Amundsen Sea Polynya^23^ and in the Ross Sea^24,25^. It has been observed to form massive aggregates at the colony stage and sink rapidly out of the ocean surface and thus has been considered to be a more efficient exporter of carbon compared to diatoms^24^.

Two mixotrophic/phagotrophic dinoflagellate OTUs were identified as key negative contributors to the geographical variability of NCP (OTU_238) and NCP/POC (OTU_318) (Fig. 2), suggesting these OTUs are likely heterotrophic. Studies in the WAP have mostly focused on large macrozooplankton grazers such as copepods, krill and salps^26,27^. In contrast to large grazers enhancing NCP by packing organic matter into rapidly-sinking fecal pellets, unicellular protistan microzooplankton are more likely to reduce carbon export through surface remineralization^28^. Microzooplankton are believed to represent an efficient top-down control on phytoplankton growth due to their short generation time and rapid reproductive cycles. With high growth and ingestion rates^29^, these small grazers could efficiently terminate an algal bloom or direct organic carbon production to DOC thereby reducing export. OTU_238 belongs to a recently-identified kleptoplastic dinoflagellate group (Supplementary Table S4)^30^, known to prey on *Phaeocystis* and retain their chloroplasts for photosynthesis^31^. Such behavior further complicates interpretation of HPLC pigments^32^. This group of dinoflagellates is abundant in Antarctic waters and sea ice^30,32,33^. Our results suggest that they may be important controls on NCP. NCP/POC was negatively correlated to the presence of dinoflagellate OTU_318, which belongs to the genus *Gyrodinium* (Supplementary Table S4) (Fig. 2B). *Gyrodinium* have been observed to ingest diatoms much larger than their own body size and may curtail diatom blooms^34,35^. A negative relationship between HPLC dinoflagellates and NCP/POC was also observed in the Australian sector of the Southern Ocean^10^.

At the higher taxonomic level (supergroup), NCP and NCP/POC were also negatively correlated with Rhizaria (r^2^ = 0.29, *p* = 0.01; r^2^ = 0.20, *p* = 0.04, respectively), a group of amoeboid heterotrophic protists with members known to feed on diatoms as well as other protists and bacteria^36^. Foraminifera, the protists known to carry autotrophic endosymbionts, are also within this supergroup. The ecology and physiology of Rhizaria are not well understood and are not detected by traditional HPLC analyses due to their lack of pigments. However, the recent discovery of their significant contribution to plankton diversity and abundance worldwide^11,37^ suggests that they must serve an important ecological function.

**Figure 3:**
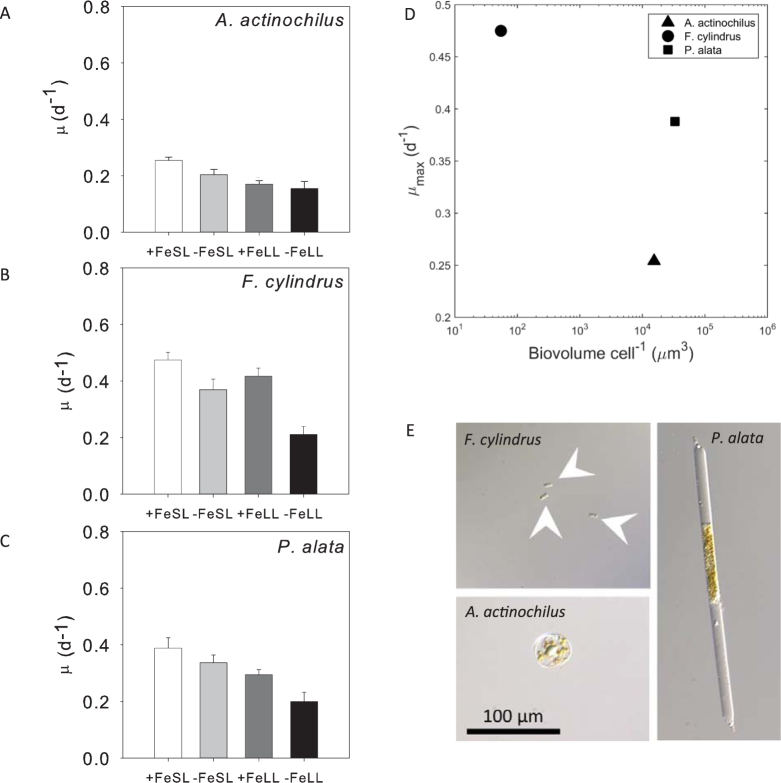
Comparison of specific growth rates and cell biovolume. Three diatom isolates collected from the WAP, *F. cylindrus* (**A**), *P. alata* (**B**), and *A. actinochilus* (**C**) a strain closely related to *Stellarima*, were maintained at 4 °C at either low light (10 μmol photons m^−2^ s^−1^) or saturating light (90 μmol photons m^−2^ s^−1^), high iron (1370 nmol L^−1^) or low iron (3.1 nmol L^−1^). The controlled culture conditions include high iron and saturating light (+FeSL), low iron and saturating light (−FeSL), high iron and low light (+FeLL), and low iron and low light (−FeLL). *F. cylindrus* has higher maximum growth rates than the other two isolates, but its biovolume is more than two orders of magnitude lower than the other two strains (**C**). Differential interference contrast images showing the cell size difference (**D**). All the images are on the same scale bar with white arrows pointing to *F. cylindrus* cells.

We further investigated the potential impact of phytoplankton species-specific growth rates and size on their relationships to carbon export potential using cultured diatom isolates recently obtained from the WAP region. With the caveat that growth rates measured under laboratory conditions may not necessarily reflect *in situ* growth rates, we found that absolute growth rates alone cannot explain the spatial variability in our observations. The distribution of *F. cylindrus* does not correlate to NCP or NCP/POC, yet its maximum growth rate in culture is significantly higher (P < 0.05) than *Proboscia alata* and *Actinocyclus actinochilus* (a strain closely related to *Stellarima*), which belong to or are closely related to the identified key diatom OTUs that are best predictors of NCP. Under varying Fe and light availability growth conditions (Fig. 3), *P. alata* and *A. actinochilus* display comparable reductions in relative growth rates to *F. cylindrus*. Yet, even though the cellular growth rates under the examined conditions were comparable between the three diatoms (i.e., varied by less than 2-fold), they are significantly different in size. The biovolume per cell for *P. alata* and *A. actinochilus* are more than two orders of magnitude larger than that of *F. cylindrus* (Fig. 3). Large size could directly enhance the sinking rates of phytoplankton cells and lead to higher export, or uncouple them from their slow-growing grazers. The extent of decoupling could also result from the differences in the response time of phytoplankton once environments are favorable, a term which is not necessarily related to absolute growth rates^38^. Phytoplankton species can be characterized by two distinct life strategies – a persistence strategy and a boom-and-bust strategy^39^. Species with a persistent life strategy are better at stress tolerance, such as *F. cylindrus* known to tolerate low temperature, photoinhibition and iron stress^40,41^. Under stable conditions, they dominate the environment in abundance, but with relatively stable growth rates they are under strong grazing control. Conversely, once the environment becomes favorable, boom-and-bust species with high growth rates can quickly outpace grazer growth and contribute to more efficient export.

**Figure 4:**
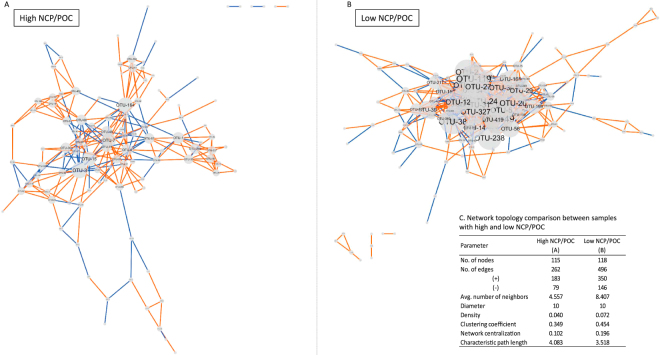
The co-occurrence network interactions of eukaryotic phytoplankton and protozoan. Data sets are divided into two groups, one with high NCP/POC (**A**) and one with low NCP/POC (**B**). The edges between different nodes (OTUs) represent strong and significant connections (SparCC correlations with magnitude > 0.75 and p-values > 0.05). Isolated nodes are not shown in the network graph. Blue edges represent negative connections and orange one represent positive connections. The nodes and OTU labels are scaled by their degrees - the number of neighbors they are interacting with. The small groups of nodes (upper right in (**A**) and lower left in (**B**)) are isolated clusters not connected to the central network. (**C**) Table reporting the network topology comparison. Networks are plotted using Cytoscape v3.4.0 (http://www.cytoscape.org).

Other than the effects of biomass, size and phytoplankton growth rates, species interactions and particularly grazing are considered regulators of carbon export. To investigate the influence of interactions on the carbon export potential, we performed a co-occurrence network analysis using SparCC (sparse correlation for compositional data^42^) on two equally divided subsets of 18 S OTUs data based on their NCP/POC values (Fig. 4). The topological features of the networks^43^ provide insights into putative plankton interactions and niche partitioning of diverse microbes in various environments^14,44,45^. In brief, the average number of connected neighbors, or network density in the normalized version (0 to 1), measures the number of neighbors a node is connected with; the network centralization describes to which extent certain nodes are highly connected to others (e.g., star-shaped network has high centralization); the clustering coefficient measures the degree of clustering between nodes, calculated as ratio of observed vs. maximum number of edges given the number of neighbors; and the characteristic path length is the average shortest path length (number of steps) to connect two nodes. The detailed properties of the low and high NCP/POC networks are summarized in Fig. 4C. Although the sample size is relatively small (i.e., 11 stations for each group), low and high NCP/POC sites are clearly different. Despite having a similar number of nodes, the low NCP/POC communities, compared to the high NCP/POC communities, are characterized by almost double the number of edges (496 vs. 262), both for positive (co-presence) and negative (e.g., mutual exclusion) interactions, as well as higher average number of connected neighbors and higher network density, higher network centralization, higher clustering coefficient, and shorter characteristic path lengths. All these features suggest that the low NCP/POC communities are more tightly coupled than the high NCP/POC communities. This may support the general paradigm that more tightly coupled ecosystems (e.g., coupling between microzookplankton grazers and phytoplankton), are less efficient exporters of carbon. Particularly, microzooplankon grazing could reduce export efficiency through longer food webs, lower trophic efficiencies, and the breakdown of large particles, enhancing organic matter remineralization. In the networks presented in Fig. 4, predator-prey coupling could be positive or negative depending on whether the system is bottom up or top down controlled. In light of the central role grazing plays in carbon fluxes, future network analyses should also include macrozooplankton.

There are several caveats to our study. A lack of steady-state in the mixed-layer carbon budget may blur some of the relationships. Normalizing NCP to POC provides us with a qualitative approach to evaluate how productive the ecosystem is for a given amount of biomass at the ocean surface. However, POC reflects an amalgam of detrital and living organic matter. Our normalization only partly accounts for the multitude of factors that are believed to influence the carbon export potential, including the proportion of detrital matter, aggregation, physiological status, plankton migration and many other processes. These factors vary as a function of the stage of the plankton ecosystem over time (e.g. blooming vs. senescence), which is not taken into account with our normalization. Uncertainty in the ratios of net community O_2_ production to CO_2_ uptake could affect our oxygen-derived calculations that assume a quotient of 1.4.^46^ have found that the PQ at the WAP may be closer to 1. While regional variation in the PQ would affect the statistical analysis, a constant bias in the quotient would not impact our conclusion. Another well-known issue is that the 16 S and 18 S reads are different from taxa-specific cell counts especially considering the variability of rDNA copy numbers per cell. However, total rDNA could possibly be an indicator of taxa-specific biomass as rDNA copy numbers generally increase with cell biovolumes across different phytoplankton species^47,48^. Our network and correlation analyses should also be interpreted with caution because 1) they do not necessarily reflect direct causal ecological relationships, 2) they may result from niche differentiation and interactions other than predation, including mutualism, competition, and parasitism, and 3) could in some cases be fortuitous or symptomatic of other abiotic and biotic factors not captured by our analyses, such as higher trophic level activity (e.g., macrozooplankton grazing), aggregation, and bacterial activity. For example, the low NCP/POC regions are mostly in the stable open ocean environments that are more homogeneous, resulting in less niche differentiation and more correlations between community members^44^. In contrast, more niche partitioning and fewer correlations are anticipated in high NCP/POC regions associated with the dynamic and heterogeneous conditions on the continental shelf and at the ice edge. Finally, a greater proportion of net community carbon production could be in the form of DOC in tightly coupled communities (in lower NCP/POC ecosystems). Overall, it will be important to further investigate these results and validate them with estimates of *in situ* growth rates, photophysiology, microzooplankon growth and grazing rates, and relations to other biotic and abiotic components of the ecosystem.

## Conclusion

This study explored the rich genomic complexity of polar ecosystems in the context of net community production, further untangling the intricate interplay of plankton community characteristics critical to carbon fluxes at the ocean surface by selecting through statistical inferences candidate key species and interactions. Within the diverse plankton communities of the WAP, as few as 2 to 3 key OTUs can explain a significant proportion of the spatial variability in carbon export potential during our observational period. Microzooplankton (i.e., dinoflagellates and Rhizaria) also arose as important negative predictors of carbon export potential, likely due to enhanced organic matter remineralization at the ocean surface. Using specific OTUs, as opposed to broad functional groups, substantially improved our ability to explain NCP variance from 35% to 92%, and NCP/POC variance from 16% to 76%. These results suggest that the PFT currency needs to be adjusted based on the biogeochemical process or element under study. As an example, diatoms require silica, and their biogeography is strongly delineated by Si availability. However, this functional grouping based on Si requirements does not necessarily translate into a cohesive group when pertaining to carbon export potential^39^. In our study, the most dominant group OTU_4 (i.e., *Fragilariopsis*) was found not to be a good predictor of net community production. Future studies of interannual variability in this and other regions of the Southern Ocean will be critical to assess the generality of our observed patterns. However, if our results are an accurate reflection of other regions of the Southern Ocean, functional group level studies should be interpreted with caution as the relation of organic carbon fluxes to taxonomy is more nuanced than currently depicted.

## Materials and Methods

We derive NCP from the ratio of oxygen (O_2_) to the inert gas argon (Ar). The biological O_2_ supersaturation can be estimated from O_2_/Ar because O_2_ and Ar have similar solubility properties. The O_2_/Ar method is suitable to explore the relation of NCP to plankton assemblages because the O_2_ residence time in this study is comparable to plankton community turnover time. The residence time of O_2_ at the ocean surface is calculated as the MLD divided by the piston velocity. For a mixed layer depth ranging from 11 to 22 m (mean 13 m) in sampled stations, with the gas exchange piston velocity calculated at each station using the weighted 60-day wind speed^49^, the O_2_ residence time in the mixed layer of 8 to 19 days (average of 11 days) (Supplementary Table S6) is within the 1 week-1 month range of phytoplankton community succession time span at the WAP^50^. We extracted total DNA from 21 stations; PCR amplified and sequenced the hypervariable V4 region of the 18 S marker gene. Based on the DNA phylogenetic information, we examined how community structure influences carbon export potential and efficiency across taxonomic ranks. In order to account for the biomass effect, NCP was normalized to POC to infer the carbon export efficiency^10,22^.

### Sampling

During the annual Palmer LTER cruise from Jan to early Feb in 2014 aboard the R/V *Laurence M. Gould*, discrete samples for POC, chlorophyll, DNA and RNA were collected at each station in the WAP region. Surface seawater from the vessel seawater supply line was gently vacuum filtered through a 0.2 µm pore size Millipore Supor filters. Each filter was then preserved immediately in 1 ml of RNA-later and stored at −80 °C. Surface seawater temperature, salinity, oxygen, fluorescence, transmissometer beam Cp and NCP were measured underway from the ship’s clean flow-through seawater line system.

### Underway NCP_(O2/Ar)_ by EIMS

The biological O_2_ supersaturation at the ocean surface was calculated as:

1 $${\rm{\Delta }}({O}_{2}/Ar)=[\frac{({O}_{2}/Ar)sample}{({O}_{2}/Ar)sat}-1]\times \,100 \% $$

ΔO_2_/Ar was measured underway from the ship’s flow-through seawater line by equilibrator inlet mass spectrometry (EIMS) as described in^9^. Assuming steady state and neglecting vertical mixing across the mixed layer boundary, NCP in units of mmol O_2_ m^−2^ day^−1^ was calculated as:

2 $$NCP=k\rho [{O}_{2}]sat\,{\rm{\Delta }}({O}_{2}/Ar)$$

where *ρ* is mixed layer density (kg m^−1^), and *k* is the gas transfer velocity for O_2_ (m day^−1^) estimated from NCEP/NCAR daily reanalysis winds (Supplementary Table S6) and a wind speed parameterization following^51^ and a gas exchange weighting as described in^49^. Mixed layer depth was determined using a density threshold ∆σθ = 0.03 kg m-3 from CTD profiles following^52^ (Supplementary Table S6). NCP for O_2_ was then converted to carbon (mmol C m^−2^ day^−1^) using O_2_/C = 1.4^53^. NCP is averaged over 10 min at the time point of water collection from the ship’s flow-through seawater line.

A recent study has shown that the NCP_(O2/Ar)_ measured in this region is highly correlated (r^2^ = 0.83) with the NCP calculated from the seasonal DIC drawdown^54^.

### Assumption of steady-state

Under steady-state or quasi-steady-state, NCP approximates carbon export out of the mixed layer because the inventory change of organic carbon (POC + DOC) in the mixed layer is negligible compared to NCP. In the Southern Ocean, DOC production is a small fraction of NCP^55,56^. In order to assess whether the assumption of steady-state is valid in our study region, we estimated the change in POC inventory over the days prior to ship sampling. To that end, we first estimated the time-derivative of chlorophyll between the time of sampling and 8 days earlier using MODIS ocean color data at 8 days 9 km resolution. We then converted Chl to POC using an empirical relationship derived from *in situ* Chl and POC (bottle measurements) data measured at the Palmer LTER (see Supplementary Fig. S3). This analysis resulted in only 4 comparisons because of cloud cover and should therefore be interpreted with caution. The estimated time-derivative of the POC inventory over the days prior to ship sampling was on average 18% of the NCP measurements, consistent with a major portion of the NCP being exported out the mixed layer. However, mismatch between NCP and carbon export production has been observed in other regions^57^. Under most conditions, NCP reflects an upper bound on carbon export, or an export potential^58^.

### DNA extraction and amplicon library construction

In the lab, from each sampled station one filter was first split in two halves, one for DNA extraction and the other for later RNA studies. 500 µl of the RNAlater from the original tube was loaded onto an Amicon 10 k column and the column was spun for 15 minutes at 14,000 g to get rid of most of the RNAlater and concentrate cells suspended in RNAlater. The resulting solution (~50 µl) was then added back to the one half filter. The cells on filter were lysed by bead-beating using 0.2 g of zirconium beads in 400 µl of buffer AP1 (Qiagen). DNA was extracted and purified using Qiagen DNeasy Plant Mini kit following the manufacture’s instruction. 18 S (eukaryotic) rDNA gene fragments were amplified by PCR using V4 primer sets: 18 S forward (5′- CCAGCASCYGCGGTAATTCC-3′) and reverse (5′-ACTTTCGTTCTTGAT-3′), modified from^59^ to avoid mismatch with haptophytes. Amplicons from each sample were tagged using dual index fusion primers^60^ and a “heterogeneity spacer”^61^ was used to improve sequencing quality. The “heterogeneity spacer” is a 0–5 bp of spacer added to the index sequence in order to allow different samples be sequenced out of phase and thus improve the amplicon library sequencing quality. Each PCR reaction (25 µl) consisted of 1U of Platinum Taq DNA Polymerase High Fidelity (Invitrogen), 1 × High Fidelity Buffer, 200 µM dNTPs, 2 mM MgSO_4_, 0.2 µM each primer, and 5–30 ng extracted environmental DNA template. PCR was conducted with an initial activation step at 94 °C for 3 min, followed by 30 three-step cycles consisting of 94 °C for 30 s, 57 °C for 30 s and 72 °C for 1 min, and a final extension step of 72 °C at 10 min. PCR products in triplicates for each sample were then pooled and purified using QIAquick PCR Purification kit, and the concentration of amplicon DNA was quantified using a Qubit dsDNA assay. Amplicons from different locations were pooled in equimolar amounts (final concentration ~10 ng/µl) and submitted to Duke Institute for Genomic Sciences and Policy (IGSP) for sequencing using Illumina MiSeq. 300PE platform. The averaged read count per sample was 105,253 after demultiplexing.

### Bioinformatic analysis

Paired-end reads were assembled using PANDAseq^62^. Assembled reads (mean length 424 bp) were further processed including quality filtering and chimera checking using USEARCH, and clustered into 97% OTUs following the UPARSE pipeline^63^. Representative sequences were aligned and assigned taxonomic identity based on the SILVA rRNA database^64^ using the software package QIIME^65^.

### Stochastic search variable selection (SSVS)

We searched for a subset of predictors, i.e. relative abundance of the top 20 OTUs, best fitting NCP or NCP/POC using Stochastic Search Variable Selection (SSVS)^66^. The predictor variables were mean-centered and variance-scaled (i.e. divided by standard deviation). If the dependent variable *y*_*i*_ (NCP or NCP/POC) is assumed to be a linear function of potential predictors *x*_*i*_ (relative abundance of OTUs), the SSVS can be set up as follow:

3 $${y}_{i}| \beta ,\,{\sigma }^{2} \sim N({x}_{i}\beta +\alpha ,\,I{\sigma }^{2})$$

where *I* is an *p* × *p* identity matrix, *x*_*i*_ is a 1 × *p* vector, *β* = (*β*_1_, *β*_2_, …*β*_*p*_)^*T*^ is a *p* × 1 vector, and *y*_*i*_, *α* and *σ* ^2^ are scalars. *i* and *p* represent sample index and number of variables, respectively. *α*, *β* and *σ* ^2^ are considered unknown, with *β*_*j*_=0 indicating that variable *x*_*i*,*p*_ is the nonselected predictor. Unknown *β* has prior distribution of $(\beta  \sim {\prod }_{j=1}^{p}\{\pi {\delta }_{0}({\beta }_{j})+(1-\pi )N({\beta }_{j};\,0,\,{\psi }_{j}^{2})\})$, where *δ*_0_ denotes Dirac delta function that places all its mass at zero. Other parameters have prior distributions of π ~ *Beta*(1, 1), ${\psi }_{j}^{-2} \sim Ga({a}_{j},\,{b}_{j})$, *σ* ^−2^~*Ga*(*a*, *b*), and $\alpha  \sim N({\mu }_{0},{\sigma }_{0}^{2})$, where *a*, *b*, *a*_*j*_, *b*_*j*_, *μ*_0_, ${\sigma }_{0}^{2}$ are constants. The posterior distributions of parameters are approximated using a sequence of random samples from Markov Chain Monte Carlo (MCMC). The MCMC is run with 10000 iterations (It) to ensure convergence with the first 5000 samples discarded (burn-in = 5000). The probability of including predictor *β*_*j*_ is defined as $p({\beta }_{j})=\frac{1}{It-B}{\sum }_{k=B+1}^{It}1({\beta }_{j,k}\ne 0)$ (4), where *B* denotes burn-in. OTUs were ranked by *p*(*β*_*j*_) (Supplementary Table S2) and the top OTUs best prediction NCP and NCP/POC were selected based on a sharp drop of *p*(*β*_*j*_) after the selected OTUs.

### Diatom isolation and identification

Diatoms were isolated from the Western Antarctic Peninsula along the PalmerLTER sampling grid in 2013 and 2014. Diatom species were identified by morphological characterization and 18 S rRNA gene (rDNA) sequencing. DNA was extracted with the DNeasy Plant Mini Kit according to the manufacturer’s protocols (Qiagen). Amplification of the nuclear 18 S rDNA region was achieved with standard PCR protocols using eukaryotic-specific, universal 18 S forward and reverse primers. Primer sequences were obtained from^67^ and are as follows: 18AF 5′-AACCTGGTTGATCCTGCCAGT-3′ and 18BR 5′- TGATCCTTCTGCAGGTTCACCTAC-3′. PCR products were purified using either QIAquick PCR Purification Kit (Qiagen) or ExoSAP-IT (Affymetrix) and sequenced by Sanger DNA sequencing (Genewiz). Sequences were edited using Geneious Pro 5.6.4 software and BLASTn sequence homology searches were performed against the NCBI nucleotide non-redundant (nr) database to determine species with a cutoff identity of 98%.

### Growth conditions, physiological characteristics and biovolumes

Isolates were maintained at 4 °C in constant irradiance at intensities of either 10 μmol photons m^−2^ s^−1^ (low light) or 90 μmol photons m^−2^ s^−1^ (growth saturating light). Light intensities were chosen based on previous culture-based experiments^41,68–70^. Cultures were grown in the synthetic seawater medium, AQUIL, enriched with filter sterilized vitamin and trace metal ion buffer containing 100 μmol L^−1^ EDTA. The growth media also contained 300 μmol L^−1^ nitrate, 200 μmol L^−1^ silicic acid and 20 μmol L^−1^ phosphate. Premixed Fe-EDTA (1:1) was added separately for total iron concentrations of either 1370 nmol L^−1^ (pFe19) or 3.1 nmol L^−1^ (pFe21.7) to achieve high iron and low iron media, respectively. All media preparation and subsampling were performed under a positive-pressure, trace metal clean laminar flow hood. Cultures were grown in acid-washed 28 mL polycarbonate centrifuge tubes (Nalgene) at least in triplicates (n ≥ 3) and maintained in exponential phase by dilution. Specific growth rates of successive transfers were calculated from the linear regression of the natural log of *in vivo* chlorophyll a fluorescence using a Turner 10-AU fluorometer during 3–7 days at exponential phase. The error bars of specific growth rates (Fig. 3) were calculated as one standard error of the mean (n ≥ 3). Growth rate comparison was perfomed using one-way ANOVA and the significance was determined using Holm-Sidak pairwise multiple comparison method.

To estimate biovolumes of each diatom species, frustules were viewed using an Olympus BX61 Upright Wide Field Microscope with the differential interference contrast imaging mode and a 60X/1.42 Oil PlanApo N objective lens. Valve apical length (AL), transapical width (TW), and pervalvar height (PH) dimensions were estimated with Scion Image software (http://scion-image.software.informer.com; June 2015). Diatom biovolumes were calculated according to^71^ where pennate diatoms were modeled after elliptic prisms and centric diatoms were modeled after cylinders.

### Network analysis

All OTUs were used in the initial dataset. The rare taxa, i.e. OTUs appearing less than three times in at least 20% of the samples, were removed from the dataset. The OTU abundances were then standardized to the median sequencing depth. The dataset was then divided into two equally sized subsets based on their NCP/POC values ≥ or ≤ the median NCP/POC. For each subset the correlations between OTUs and the p-values were computed using the SparCC method^42^, which was specially designed for compositional data. The resulting correlations with magnitude >0.75 and with p-values >0.05 were selected to construct networks for each subset. The two networks were visualized using Cytoscape v3.4.0^72^. In Cyoscape, OTUs are represented as nodes. Correlations are displayed as edges with positive relationships in orange and negative ones in blue. Network properties were computed using the ‘Network Analysis’ function in Cytoscape, including diameter, density, clustering coefficient, characteristic path length, etc. The topological parameters for each node were also calculated, such as node degree (the number of edges linked to each node), betweenness centrality, and closeness centrality.

### Other statistical analyses

Forward stepwise regression was performed in JMP statistical discovery software from SAS. To reduce the number of regressors in the stepwise regression, which increase the possibility of collinearity and over-fitting, only the top 20 most dominant OTUs were included in the analysis. The best model was selected based on Akaike’s information criterion correction (AICc). AICc is suitable for small sample size (here n = 21), and it reduces over-fitting by greater penalty for increasing number of parameters included in the model. Even though significant in stepwise regression, some OTUs (Supplementary Table S3) were not included in the final multiple linear regression model because they were not selected by SSVS and each OTU only slightly improved the adjusted R^2^ of the model (≤0.05).

Partition of variation was performed on selected variables vs. NCP or NCP/POC in R (R software, Vienna, Austria) using function “varpart” in the “vegan” package. Briefly, the unique (non-overlapping) or shared (overlapping) fractions of NCP or NCP/POC variance explained by OTUs were calculated by partial regressions and then decomposing the adjusted R^2^ ^73^.

### Data availability

Sequences and associated metadata have been deposited in the NCBI Sequence Read Archive under the accession number SRP119815. Other datasets are available from the corresponding author on reasonable request.

## Electronic supplementary material

Supplementary Figures and Tables

Supplementary Table S6
