# Supplementary material for: Specific eukaryotic plankton are good predictors of net community production in the Western Antarctic Peninsula
Source: Sci Rep. 2017 Nov 1;7:14845. doi: 10.1038/s41598-017-14109-1 (PMC5665988; doi:10.1038/s41598-017-14109-1)
Supplement: Supplementary file 1 — Supplementary Figures and Tables [file 41598_2017_14109_MOESM1_ESM.pdf]

Specific eukaryotic plankton are good predictors of net community production in the Western Antarctic Peninsula

Yajuan Lin, Nicolas Cassar, Adrian Marchetti, Carly Moreno, Hugh Ducklow & Zuchuan Li

Supplementary figures and tables;

Supplementary Figure S1 Chlorophyll concentration
Supplementary Figure S2 Relative abundance (%) of OTU 4
Supplementary Figure S3 Empirical relationship of POC and Chlorophyll
Supplementary Figure S4 Monthly averaged sea ice coverage for Jan 2014
Supplementary Table S1 Correlations of 18S taxonomy at higher level (Phylum to Division) versus NCP and NCP/POC
Supplementary Table S2 Stochastic search variable selection (SSVS)
Supplementary Table S3 Stepwise regression based on AICc for selection of OTUs best predictors of NCP and NCP/POC
Supplementary Table S4 Taxonomy assignments of the representative sequences from the top 20 OTUs
Supplementary Table S5 SSVS with station 600.40 removed

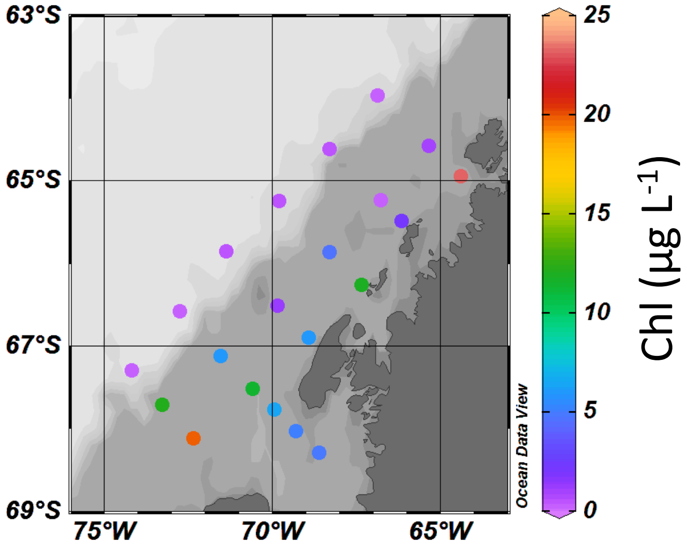

Supplementary Figure S1: Chlorophyll concentration. Map created with Ocean Data View version 4.6.3.1 (Schlitzer, R., Ocean Data View, http://odv.awi.de, 2015).

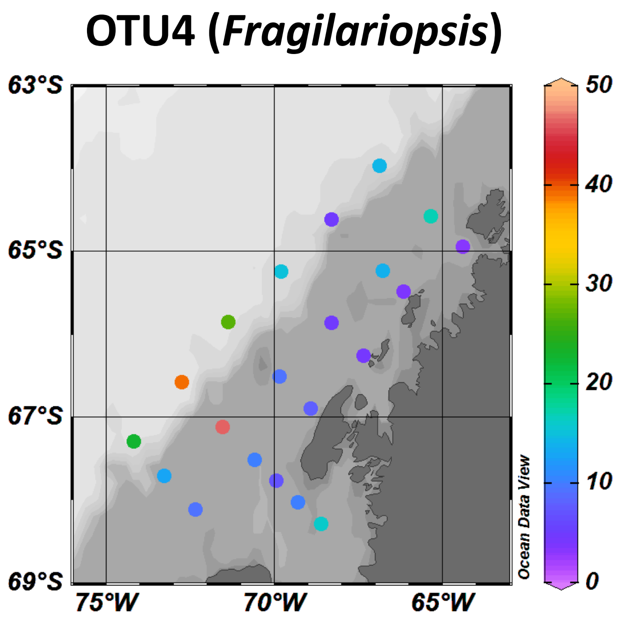

Supplementary Figure S2: Relative abundance (%) of OTU 4. OTU 4 (*Fragilariopsis*) is the most dominant OTU. Map created with Ocean Data View version 4.6.3.1 (Schlitzer, R., Ocean Data View, http://odv.awi.de, 2015).

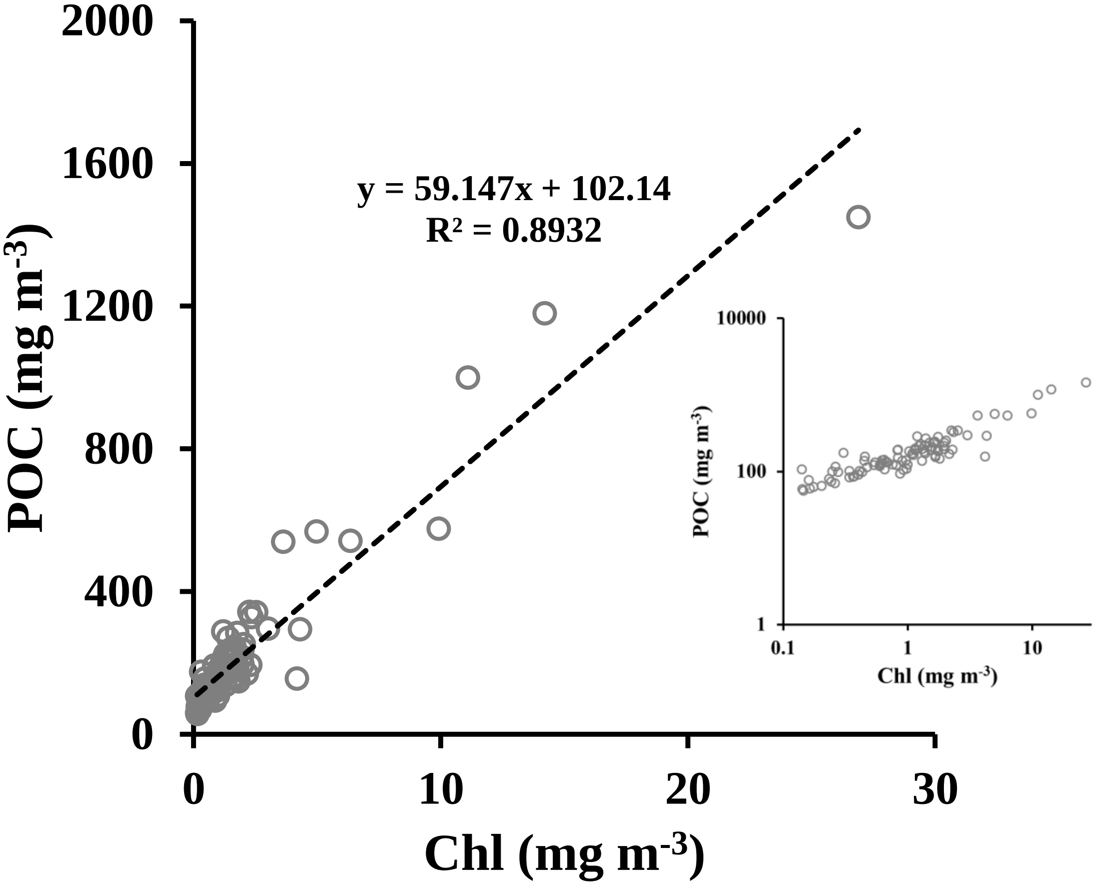

Supplementary Figure S3: Empirical relationship of POC and Chlorophyll. The relationship is derived from Palmer LTER annual cruise data 2008 – 2011 (n = 93).

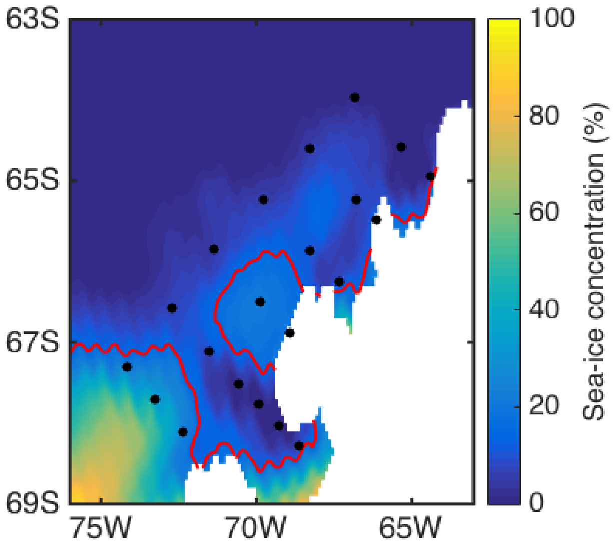

Supplementary Figure S4: Monthly averaged sea ice coverage for Jan 2014. Sea ice data was downloaded from NSIDC using Version 2 of the Bootstrap sea ice concentrations from Nimbus-7 SMMR and DMSP SSM/I-SSIMS. Ice edge in red line was defined by the 15% ice concentration contour. Map generated by MATLAB R2014a (http://www.mathworks.com/).

Supplementary Table S1: Correlations of 18S taxonomy at higher level (Phylum to Division) versus NCP and NCP/POC.

Taxa vs. NCP	Pearson		Spearman		Kendall		Stepwise (AdjR² 0.37)	
(21stn)	R	P	Rho	P	Tau	P	Coefficient	P
Dinoflagellata	-0.0486	0.8341	-0.2909	0.2002	-0.1714	0.2945		
Cryptophyta	-0.5825	0.0056	-0.7558	0.0001	-0.5429	0.0004		
Haptophyta	0.036	0.877	0.0182	0.9393	0.019	0.9287		
Rhizaria	-0.5417	0.0112	-0.674	0.0011	-0.4476	0.004		
Diatomea	0.5885	0.005	0.6727	0.0011	0.4667	0.0026	124.946	0.003
Diatoms (Pennate)	-0.265	0.2457	-0.2026	0.3768	-0.1619	0.3232		
Diatoms (Centric)	0.6864	0.0006	0.774	0.0001	0.5619	0.0002		

Taxa vs. NCP/POC	Pearson		Spearman		Kendall		Stepwise (AdjR² 0.46)	
(21stn)	R	P	Rho	P	Tau	P	Coefficient	P
Dinoflagellata	-0.0464	0.8418	-0.1143	0.6207	-0.0857	0.6119		
Cryptophyta	-0.4277	0.0531	-0.5091	0.0198	-0.3048	0.0564	-8.6497	0.0083
Haptophyta	0.5304	0.0134	0.1597	0.4875	0.1238	0.455	6.49739	0.0024
Rhizaria	-0.4455	0.043	-0.7143	0.0004	-0.4952	0.0013		
Diatomea	0.3983	0.0737	0.5922	0.0055	0.4381	0.005		
Diatoms (Pennate)	-0.1513	0.5126	-0.161	0.4839	-0.1143	0.4921		
Diatoms (Centric)	0.449	0.0412	0.6377	0.0024	0.4571	0.0033		

Supplementary Table S2: Stochastic search variable selection (SSVS).

NCP		NCP/POC	
OTU#	$p(\beta_j)$	OTU#	$p(\beta_j)$
**OTU_8**	**0.9490**	**OTU_318**	**0.3403**
**OTU_238**	**0.5790**	**OTU_3**	**0.1086**
**OTU_6**	**0.5020**	OTU_164	0.0048
OTU_15	0.1200	OTU_2	0.0024
OTU_150	0.0422	OTU_5	0.0018
OTU_5	0.0178	OTU_37	0.0014
OTU_2	0.0138	OTU_11	0.0014
OTU_11	0.0036	OTU_22	0.0012
OTU_318	0.0028	OTU_6	0.0010
OTU_3	0.0024	OTU_9	0.0008
OTU_164	0.0022	OTU_7	0.0006
OTU_10	0.0020	OTU_119	0.0006
OTU_1	0.0018	OTU_8	0.0006
OTU_119	0.0016	OTU_238	0.0004
OTU_37	0.0012	OTU_150	0.0004
OTU_13	0.0008	OTU_4	0.0002
OTU_4	0.0006	OTU_1	0.0002
OTU_7	0.0004	OTU_15	0.0002
OTU_9	0.0004	OTU_10	0.0000
OTU_22	0.0002	OTU_13	0.0000

Selected OTUs best predictors of NCP (A) and NCP/POC (B). OTUs are ranked by $p(\beta_j)$, the estimated posterior probability of the OTU to be included in the model explaining NCP or NCP/POC. OTUs in red were selected as the best predictors based on a sharp drop of $p(\beta_j)$ after the selected OTUs.

Supplementary Table S3: Stepwise regression based on AICc for selection of OTUs best predictors of NCP and NCP/POC.

OTU vs. NCP

OTU#	Taxa	AICc	R^2	AdjR^2	F Ratio	Prob>F
OTU8	*Stellarima*	214.113	0.6391	0.6201	88.730	3.69E-08
OTU6	*Proboscia*	199.388	0.8455	0.8283	31.169	3.30E-05
OTU238	novel Dino	185.726	0.9318	0.9197	21.492	0.00024
OTU15	*Thalassiosira*	182.852	0.9508	0.9385	24.291	0.00028
OTU164	*Karenia*	179.985	0.9655	0.9541	13.488	0.00281
OTU150	*Pseudo-nitzschia*	180.129	0.9732	0.9617	7.233	0.01857
OTU4	*Fragilariopsis*	179.892	0.9804	0.9698	4.801	0.04726

OTU vs. NCP/POC

OTU#	Taxa	AICc	R^2	AdjR^2	F Ratio	Prob>F
OTU318	*Gyrodinium*	38.2141	0.5502	0.5266	36.202	0.00002
OTU3	*Phaeocystis*	25.7071	0.7860	0.7622	22.261	0.00027
OTU6	*Proboscia*	22.9821	0.8409	0.8128	9.565	0.00743
OTU164	*Karenia*	22.8924	0.8690	0.8363	8.562	0.01043
OTU9	*Picobiliphyte*	21.5283	0.9015	0.8687	4.941	0.04202

OTUs in grey were not included in the final multiple linear regression model (Fig. 2) because they were not selected by SSVS and for each OTU the improvement of accumulated r^2 was small to negligible (≤ 0.05).

Supplementary Table S4: Taxonomy assignments of the representative sequences from the top 20 OTUs.

(A) Based on SILVA 119 database and SILVA Incremental Aligner

OTU#	Identity	Quality	Silva Taxa
OTU_4	96.1538	99	Eukaryota;SAR;Stramenopiles;Ochrophyta;Diatomea;Bacillariophytina;Bacillariophyceae;Fragilariopsis;
OTU_1	99.2188	99	Eukaryota;SAR;Stramenopiles;Ochrophyta;Diatomea;Bacillariophytina;Mediophyceae;Chaetoceros;
OTU_3	100	100	Eukaryota;Haptophyta;Prymnesiophyceae;Phaeocystis;
OTU_238	97.1354	97	Eukaryota;SAR;Alveolata;Dinoflagellata;Dinophyceae;SL163A10;
OTU_7	98.1771	98	Eukaryota;SAR;Alveolata;Dinoflagellata;Dinophyceae;Gymnodiniphycidae;Gyrodinium;
OTU_5	88.3544	97	Eukaryota;SAR;Rhizaria;Cercozoa;Thecofilosea;
OTU_119	98.4375	98	Eukaryota;SAR;Alveolata;Dinoflagellata;Dinophyceae;Gymnodiniphycidae;Kareniaceae;Karlodinium;
OTU_6	88.9175	95	Eukaryota;SAR;Stramenopiles;Ochrophyta;Diatomea;Coscinodiscophytina;Rhizosolenids;Proboscia;
OTU_318	96.3542	96	Eukaryota;SAR;Alveolata;Dinoflagellata;Dinophyceae;Gymnodiniphycidae;Gyrodinium;
OTU_2	98.939	99	Eukaryota;Cryptophyceae;Cryptomonadales;
OTU_37	97.3958	97	Eukaryota;SAR;Alveolata;Dinoflagellata;Dinophyceae;Gymnodiniphycidae;
OTU_164	97.9167	98	Eukaryota;SAR;Alveolata;Dinoflagellata;Dinophyceae;SCM16C67;
OTU_13	86.3049	96	Unclassified;
OTU_10	96.6234	98	Eukaryota;SAR;Stramenopiles;Ochrophyta;Diatomea;Bacillariophytina;Bacillariophyceae;Navicula;
OTU_15	90.1809	97	Eukaryota;SAR;Stramenopiles;Ochrophyta;Diatomea;Bacillariophytina;Mediophyceae;Thalassiosira;
OTU_9	80.6202	87	Unclassified;
OTU_11	97.6744	99	Eukaryota;SAR;Stramenopiles;Ochrophyta;Diatomea;Bacillariophytina;Mediophyceae;Thalassiosira;
OTU_22	97.9167	98	Eukaryota;SAR;Alveolata;Dinoflagellata;Dinophyceae;Gymnodiniphycidae;Gymnodinium clade;Gymnodinium;
OTU_8	86.8557	95	Eukaryota;SAR;Stramenopiles;Ochrophyta;Diatomea;Coscinodiscophytina;Stellarima;
OTU_150	96.9152	98	Eukaryota;SAR;Stramenopiles;Ochrophyta;Diatomea;Bacillariophytina;Bacillariophyceae;Pseudo-nitzschia;

(B) based on NCBI Blastn top hit

OTU#	Blastn Taxa		E value	Identity %
OTU_4	Fragilariopsis cylindrus	Eukaryota; Stramenopiles; Bacillariophyta; Bacillariophyceae;Bacillariophycidae; Bacillariales; Bacillariaceae;	0	100
OTU_1	Chaetoceros neogracile	Eukaryota; Stramenopiles; Bacillariophyta; Coscinodiscophyceae;Chaetocerotophycidae; Chaetocerotales; Chaetocerotaceae;	0	99
OTU_3	Phaeocystis antarctica voucher NCMA:CCMP1374	Eukaryota; Haptophyceae; Phaeocystales; Phaeocystaceae;	0	100
OTU_238	Dinophyceae sp. W5-1	Eukaryota; Alveolata; Dinophyceae; unclassified Dinophyceae	0	98
OTU_7	Gyrodinium rubrum	Eukaryota; Alveolata; Dinophyceae; Gymnodiniales; Gymnodiniaceae;	0	100
OTU_5	Cryothecomonas sp. APCC MC5-1Cryo	Eukaryota; Rhizaria; Cercozoa; Thecofilosea; Cryomonadida;	0	99
OTU_119	Karlodinium veneficum	Eukaryota; Alveolata; Dinophyceae; Gymnodiniales; Kareniaceae;	0	98
OTU_6	Proboscia inermis	Eukaryota; Stramenopiles; Bacillariophyta; Coscinodiscophyceae;Rhizosoleniophycidae; Rhizosoleniales; Rhizosoleniaceae;	0	100
OTU_318	Gyrodinium helveticum	Eukaryota; Alveolata; Dinophyceae; Gymnodiniales; Gymnodiniaceae;	0	98
OTU_2	Geminigera cryophila	Eukaryota; Cryptophyta; Pyrenomonadales; Geminigeraceae;	0	99
OTU_37	Gyrodinium cf. spirale AR-2015	Eukaryota; Alveolata; Dinophyceae; Gymnodiniales; Gymnodiniaceae;	0	98
OTU_164	Karenia bidigitata	Eukaryota; Alveolata; Dinophyceae; Gymnodiniales; Kareniaceae;	0	98
OTU_13	Corethron inerme	Eukaryota; Stramenopiles; Bacillariophyta; Coscinodiscophyceae;Corethrophycidae; Corethrales; Corethraceae;	0	97
OTU_10	Navicula cari	Eukaryota; Stramenopiles; Bacillariophyta; Bacillariophyceae;Bacillariophycidae; Naviculales; Naviculaceae;	0	99
OTU_15	Gyrosigma acuminatum	Eukaryota; Stramenopiles; Bacillariophyta; Bacillariophyceae; Bacillariophycidae; Naviculales; Pleurosigmataceae;	1e-163	94
OTU_9	Picobiliphyte sp. MS584-22	Eukaryota; Picozoa; unclassified Picozoa	0	98
OTU_11	Thalassiosira tumida	Eukaryota; Stramenopiles; Bacillariophyta; Coscinodiscophyceae;Thalassiosirophycidae; Thalassiosirales; Thalassiosiraceae;	0	100
OTU_22	Warnowia sp. 5 AR-2015	Eukaryota; Alveolata; Dinophyceae; Gymnodiniales; Warnowiaceae;	0	99
OTU_8	Stellarima microtrias	Eukaryota; Stramenopiles; Bacillariophyta; Coscinodiscophyceae;Coscinodiscophycidae; Coscinodiscales; Coscinodiscaceae;	0	100
OTU_150	Pseudo-nitzschia australis	Eukaryota; Stramenopiles; Bacillariophyta; Bacillariophyceae; Bacillariophycidae; Bacillariales; Bacillariaceae;	0	98

Re-analyses with high value stations removed:

1) Removing station 600.40, the station displaying the highest NCP and dominated by OUT_8: in SSVS OTU_8 was still a strong predictor of NCP, and the OTU based approach explained a significantly higher percentage of variance in NCP (bulk diatoms r^2 = 0.45, OTUs r^2 = 0.82), and NCP/POC (bulk diatoms r^2 = 0.15, OTUs r^2 = 0.76).

Supplementary Table S5: SSVS with station 600.40 removed.

NCP	
**OTU#**	***p(β$_j$)***
**OTU6**	**0.1178**
**OTU8**	**0.0890**
**OTU238**	**0.0740**
OTU5	0.0332
OTU11	0.0322
OTU150	0.0176
OTU2	0.0162
OTU15	0.0086
OTU1	0.0054
OTU318	0.0038
OTU37	0.0036
OTU119	0.0028
OTU3	0.0020
OTU13	0.0008
OTU4	0.0006
OTU164	0.0006
OTU7	0.0004
OTU10	0.0004
OTU9	0.0004
OTU22	0.0002

NCP/POC	
**OTU#**	***p(β$_j$)***
**OTU_318**	**0.2153**
**OTU_3**	**0.0550**
OTU_164	0.0072
OTU_238	0.0052
OTU_2	0.0018
OTU_6	0.0014
OTU_11	0.0010
OTU_37	0.0008
OTU_8	0.0008
OTU_4	0.0006
OTU_9	0.0006
OTU_22	0.0006
OTU_7	0.0004
OTU_5	0.0004
OTU_15	0.0004
OTU_1	0.0002
OTU_119	0.0002
OTU_10	0.0002
OTU_13	0.0002
OTU_150	0.0002

2) Removing station 200.100, the station with the highest OTU_4 (*Fragilariopsis*): OTU_4 was still the most abundant OTU (averaged relative abundance 12.5%) and it was not significantly correlated with NCP ($r^2 = 0.08$, $p > 0.1$), or NCP/POC ($r^2 = 0.04$, $p > 0.1$).
